# The Gut-Liver Axis in Health and Disease: The Role of Gut Microbiota-Derived Signals in Liver Injury and Regeneration

**DOI:** 10.3389/fimmu.2021.775526

**Published:** 2021-12-10

**Authors:** Zhipeng Zheng, Baohong Wang

**Affiliations:** State Key Laboratory for Diagnosis and Treatment of Infectious Diseases, National Clinical Research Center for Infectious Diseases, Collaborative Innovation Center for Diagnosis and Treatment of Infectious Diseases, The First Affiliated Hospital, Zhejiang University School of Medicine, Hangzhou, China

**Keywords:** gut microbiota (GM), liver injury and regeneration, lipopolisaccharide (LPS), bile acid (BA), SCFA (short chain fatty acids), tryptophan metabolites, gut microbial metabolites

## Abstract

Diverse liver diseases undergo a similar pathophysiological process in which liver regeneration follows a liver injury. Given the important role of the gut-liver axis in health and diseases, the role of gut microbiota-derived signals in liver injury and regeneration has attracted much attention. It has been observed that the composition of gut microbiota dynamically changes in the process of liver regeneration after partial hepatectomy, and gut microbiota modulation by antibiotics or probiotics affects both liver injury and regeneration. Mechanically, through the portal vein, the liver is constantly exposed to gut microbial components and metabolites, which have immense effects on the immunity and metabolism of the host. Emerging data demonstrate that gut-derived lipopolysaccharide, gut microbiota-associated bile acids, and other bacterial metabolites, such as short-chain fatty acids and tryptophan metabolites, may play multifaceted roles in liver injury and regeneration. In this perspective, we provide an overview of the possible molecular mechanisms by which gut microbiota-derived signals modulate liver injury and regeneration, highlighting the potential roles of gut microbiota in the development of gut microbiota-based therapies to alleviate liver injury and promote liver regeneration.

## Introduction

The liver has an outstanding regenerative capacity (1). Liver regeneration is a well-orchestrated biological process that depends on a large series of signals. Following different types of damage, the remnant liver initiates different types of reprogramming events, which activate different progenitor cells to replace injured cells (2). The regenerating liver undergoes numerous adaptive responses in gene expression, growth factor production, and morphological structure, which have been extensively described (1, 3). The essential gene expressions required for liver regeneration cover cytokine, growth factor, and metabolic, which interact with each other and fine-tune regenerative responses to maintain hepatic homeostasis according to body demands (4). The classical mechanisms of liver regeneration focus on signaling pathways within the liver. However, recent studies have evidenced that commensal gut microbiota plays local and systematic roles in tissue repair and regeneration (5). Therefore, it is extremely important to describe the interaction between gut microbiota and liver in the regulation of liver injury and regeneration.

Through the portal vein, the liver is constantly exposed to bacterial components and gut microbial metabolites. Lipopolysaccharide (LPS) is a cell wall component of gram-negative bacteria, and a mild release of LPS from the gut can stimulate liver regeneration and tissue repair (6–8). Besides, gut microbial metabolites, such as bile acids, short-chain fatty acids (SCFAs), and tryptophan metabolites, affect host metabolism and immune system (9, 10), which may indirectly influence liver injury and regeneration. Herein, the review aims to elucidate the potential molecular mechanisms that gut microbiota interacts with the liver from the perspective of injury and regeneration, which may provide valuable clues to develop gut microbiota-based therapies for liver diseases.

## Gut Microbiota and Liver Diseases

Gut microbiota dysbiosis has been found in liver diseases with distinct etiologies, including acute liver injury, viral hepatitis, non-alcoholic fatty liver disease (NAFLD), alcohol-related liver disease, autoimmune hepatitis (AIH), primary biliary cholangitis (PBC), and primary sclerosing cholangitis (PSC) (11, 12). (**Table 1**) Diverse liver diseases undergo a similar pathophysiological process, in which the damaged liver needs repair and gut microbiota may play an important role.

**Table 1:** Gut dysbiosis in liver diseases.

Liver disease	Organism	Reference
**Hepatitis B**	*Bifidobacteria*/Enterobacteriaceae ↓	(13)
**NASH**	*Bacteroides* ↑, *Prevotella* ↓, *Ruminococcus* ↑	(14)
**NAFLD**	High-alcohol-producing *Klebsiella pneumoniae* ↑	(15)
**AIH**	*Veillonella dispar* ↑	(16)
**PSC**	*Veillonella*↑	(17)
**PBC**	Microbial diversity ↓, *Faecalibacterium* ↓	(18)
**Liver cirrhosis**	Enterobacteriaceae ↑, Streptococcaceae ↑, Lachnospiraceae ↓	(19)
**Liver cirrhosis with HE**	*Enterococcus*↑, *Veillonella* ↑, *Megasphaera* ↑ *Burkholderia* ↑, *Roseburia* ↑	(20)
**ACLF**	Lachnospiraceae + Ruminococcaceae + Veillonellaceae + Clostridiales Cluster XIV/Enterobacteriaceae + Bacteroidaceae ↓	(21)
**HCC**	Actinobacteria ↑, Verrucomicrobia ↓	(22)

In pre-cirrhotic liver disease, the gut microbiota has changed. The ratio of *Bifidobacteria*/Enterobacteriaceae is gradually reduced in healthy individuals, hepatitis B virus carriers, patients with chronic hepatitis B, and patients with decompensated cirrhosis (13), suggesting that the alteration of gut microbiota is associated with disease progression in hepatitis B patients. In NASH patients, the relative abundance of *Bacteroides* significantly increases and *Prevotella* decreases, whereas the relative abundance of *Ruminococcus* is higher in F ≥ 2 fibrosis patients (14). A recent study finds that high-alcohol-producing *Klebsiella pneumoniae* is associated with almost 60% of NAFLD, and high-alcohol-producing *Klebsiella pneumoniae* supplement by oral gavage induces NAFLD in mice (15), suggesting that endogenous alcohol production by gut microbiota drives NAFLD in some cases. In addition, a gut microbiota-based metagenomic signature can be used to distinguish mild and moderate NAFLD from advanced fibrosis (23). For AIH, increased abundance of *Veillonella dispar* is linked to disease severity, and the combination of *Veillonella*, *Lactobacillus*, *Oscillospira*, and *Clostridiales* discriminates AIH from controls (16). The relative abundance of *Veillonella* is also enriched in PSC compared with healthy controls and ulcerative colitis patients without liver diseases (17). Microbial diversity is significantly reduced in PBC patients, and *Faecalibacterium* is further decreased in gp210-positive than gp210-negative PBC patients (18). These correlations between pre-cirrhotic liver diseases and gut microbiota indicate that the primary injury of liver diseases shape, and are shaped by, changes in gut microbiota composition.

Gut dysbiosis is more obvious in liver cirrhosis, the pathological end-stage of chronic liver disease, and can cause complications. The enrichment of potentially pathogenic bacteria Enterobacteriaceae and Streptococcaceae and the reduction of beneficial bacteria Lachnospiraceae in patients with liver cirrhosis have a positive and negative correlation with the Child-Turcotte-Pugh score, which is used to assess the severity of cirrhosis based on five clinical parameters, respectively (19). Quantitative metagenomics reveals that a combination of 15 optimal microbiota-targeted gene markers (NLF009_gene_80134, H16_gene_75905, et al.) discriminates liver cirrhosis patients from healthy individuals with a training AUC value of 0.918 and a validating AUC value of 0.836 (24). Decompensated liver cirrhosis could be accompanied by a severe central nervous system, namely hepatic encephalopathy (HE). There is no difference in stool microbiota between HE and no-HE patients, but mucosal microbiota obtained by sigmoidoscopy is different with increased abundance of *Enterococcus*, *Veillonella*, *Megasphaera*, and *Burkholderia* and decreased abundance of *Roseburia* in HE patients (20). The further development of decompensated liver cirrhosis towards acute-on-chronic liver failure (ACLF). The cirrhosis dysbiosis ratio (Lachnospiraceae + Ruminococcaceae + Veillonellaceae + Clostridiales Cluster XIV/Enterobacteriaceae + Bacteroidaceae) is lower in patients who progress to ACLF and associated with an elevated risk of extra-hepatic failure and death (21). Besides, most hepatocellular carcinoma (HCC) develops in the setting of advanced liver cirrhosis. Dysbiosis of gut microbiota is associated with increased inflammation, impaired intestinal barrier, and immune system disorders, which are involved in HCC development (22). The complexity and role of gut microbiota in end-stage chronic liver disease suggest that gut microbiota modulation may be a way to deal with difficulties in liver injury and regeneration.

Consequently, these studies suggest that gut microbiota may have a significant impact on the pathophysiology of liver diseases, in which abnormal ductular responses, excessive fibrosis, and impaired innate immunity can inhibit normal regeneration and lead to liver failure or tumors. How to restore the regenerative ability of the failed liver is an essential problem to be solved in clinical scenarios. A comprehensive understanding of the underlying mechanisms may enable appropriate targets of gut microbiota-based therapies to reduce the factors that inhibit liver regeneration or directly stimulate liver regeneration.

## Gut Microbiota Dynamically Changes During Liver Regeneration

**Figure 1:**
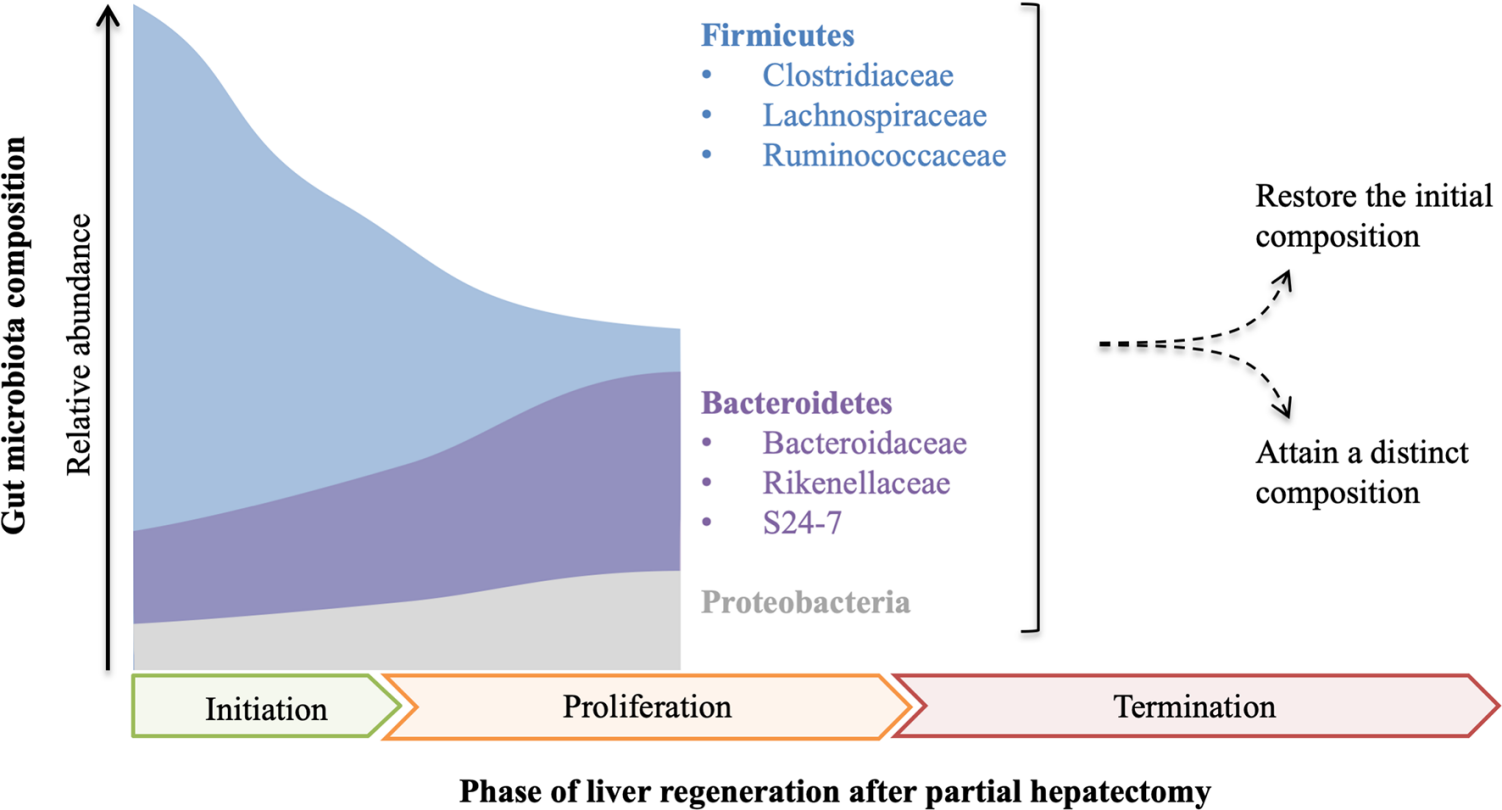
The composition of gut microbiota is fluctuant in the course of liver regeneration. After partial hepatectomy, the relative abundance of Firmicutes decreases while Bacteroidetes and Proteobacteria increase, which probably continues until the middle of the proliferation phase of liver regeneration. As proliferation and termination of liver regeneration progress, the final composition of gut microbiota remains controversial and needs further study.

The relationship between gut microbiota and liver regeneration has been studied in the animal partial hepatectomy models (**Figure 1**). Dynamic changes of gut microbiota are observed in mice from 0 hours to 9 days after partial hepatectomy (25). Partial hepatectomy leads to a distinct change in the composition of gut microbiota, early at 1 hour after partial hepatectomy, with steadily increased Bacteroidetes and decreased Firmicutes, which account for the most abundant phyla. At the family level, increased S24-7 and Rikenellaceae make up the most abundant taxa within Bacteroidetes phylum, while decreased Clostridiaceae, Lachnospiraceae and Ruminococcaceae are the most abundant representatives in Firmicutes phylum. Moreover, alteration of S24-7, Lachnospiraceae, and Ruminococcaceae is closely associated with hepatic metabolism and proliferation. The shifts of bacterial populations persist for 9 days in mice after partial hepatectomy, which almost covers all of the priming phase, proliferative phase, and termination phase of regenerating liver (25).

In the other study, fluctuating alterations of gut microbiota are observed in rats after partial hepatectomy (26). In this study, the abundance of Bacteroidetes rapidly decreases at 12 hours after partial hepatectomy, but steadily increases to the initial level at 48 hours, and then decreases to a low level again at 72 hours and lasts to the endpoint. Compared with Bacteroidetes, the alteration of Firmicutes shows a different trend. The ratio of Firmicutes to Bacteroidetes (F/B) is fluctuant throughout the process of liver regeneration. Notably, the abundance of Proteobacteria has a remarkable elevation at 48 hours after partial hepatectomy, but it almost decreases to the initial level before the endpoint. At the family level, Lachnospiraceae and Ruminococcaceae increase in 12-24 hours and 3-14 days after partial hepatectomy, but they are decreased in 30-48 hours. Furthermore, cluster analysis indicates that the composition of gut microbiota is different along with the process of liver regeneration (26).

Although the changing trends of gut microbiota are not identical in the only two published studies, which may be the consequence of different experimental designs, increased Bacteroidetes and decreased Firmicutes are recognized in the priming phase and the partial proliferative phase of liver regeneration. As the proliferation and termination of liver regeneration progress, whether the composition of gut microbiota is finally restored or attains a new state remains unclear and needs further study. These data indicate that gut microbiota has a potential influence on the regenerating liver or vice versa.

## Gut Microbiota Manipulations Affect Liver Injury and Regeneration

**Figure 2:**
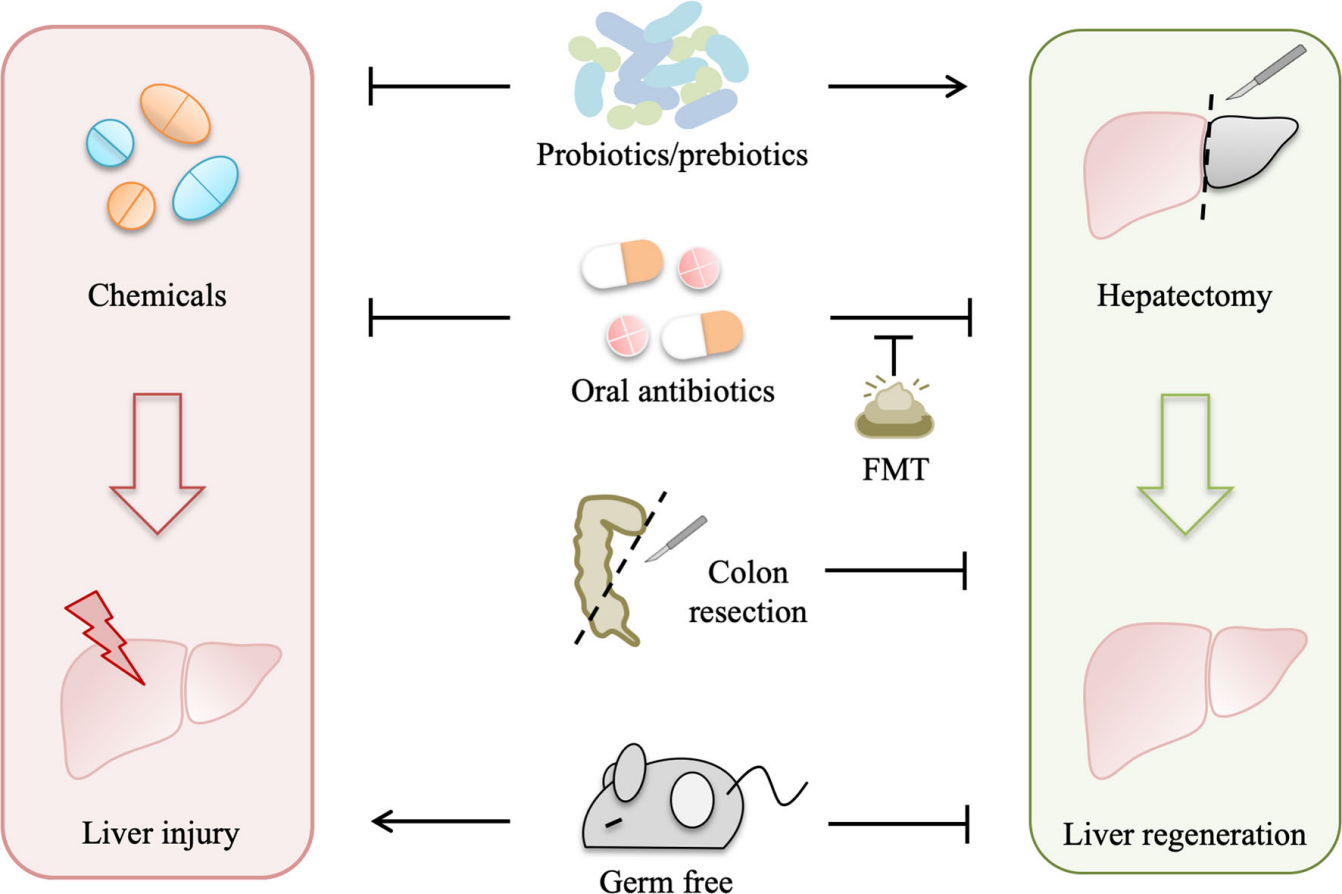
Gut microbiota manipulations affect liver injury and regeneration. Gut microbiota depletion by several approaches, including germ-free, antibiotics, and colon resection, suppresses liver regeneration to varying degrees, while fecal microbiota transplant (FMT) can normalize liver regeneration in the antibiotic-treated model, and probiotics/prebiotics can promote liver regeneration. In addition, both probiotics and oral antibiotics alleviate drug-induced acute liver injury, which is aggravated in germ-free rodents.

Gut microbiota is depleted in germ-free model and can be modulated by antibiotics, probiotics, prebiotics, such as dietary fibers (27), fecal microbiota transplant (FMT), and colon resection. All of these gut microbiota manipulations influence liver injury and regeneration (**Figure 2**).

Gut bacterial depletion using oral non-absorbable antibiotics depresses liver regeneration in rats after partial hepatectomy, and liver regeneration is also impaired in germ-free mice with partial hepatectomy (7, 8). Likewise, liver regeneration is suppressed in rats with simultaneous liver and colon partial resection (28). These studies indicate that gut microbiota is required for normal liver regeneration. Interestingly, gut microbiota participates in liver injury as well as liver regeneration. Oral antibiotics prevent liver injury induced by hepatotoxic agents, such as CCl_4_, acetaminophen, D-Gal, and alcohol (29), whereas a complete absence of gut microbiota as in germ-free rodents can exacerbate the acute liver injury (30), suggesting that gut microbiota also contributes to the pathophysiology of drug-induced liver injury.

Moreover, FMT normalizes impaired liver regeneration in rats with gut decontamination by antibiotics (26), which further manifests that normal gut microbiota plays a driving role in liver regeneration. Probiotics improve the outcome of partial hepatectomy, not only in animal experiments but also in some clinical trials (31–33). Probiotic supplement improves mitosis in the liver of rats with simultaneous 70% partial hepatectomy and colon anastomosis probably by preventing bacteria translocation (31). In hepatocellular carcinoma (HCC) patients receiving hepatic resection, preoperative and postoperative probiotics improve liver function and reduce complications (32). In another pilot study with 19 patients subjected to right hepatectomy, symbiotics can improve liver function after liver resection in the uncomplicated subgroup (33). Likewise, emerging evidence demonstrates that probiotics alleviate drug-induced liver injury in animal experiments. *Lactobacillus rhamnosus* improves liver function and ameliorates alcohol-induced liver injury in mice (34, 35). *Bifidobacterium adolescentis*, *Bacillus cereus*, and *Lactobacillus helveticus* pretreatments can modify the gut microbiota and alleviate liver injury in D-Gal-treated rats (36–38). *Akkermansia muciniphila* protects mice from immune-mediated liver injury (39).

Dynamic changes of gut microbiota during liver regeneration and the benefits of FMT and probiotics on liver injury and regeneration indicate that the crosstalk between the liver and gut microbiota is important for liver regeneration, which is probably mediated by gut microbiota-derived components and metabolites.

## Gut-Derived LPS Have Multiple Effects on Liver Injury and Regeneration

Gut-derived LPS, produced by enteric gram-negative bacteria, are continually presented to the liver (6), and low-grade portal venous LPS can be cleared by the liver (40). Under normal conditions, gut-derived LPS can be phagocytized and detoxified by Kupffer cells in the liver reticuloendothelial system (RES) (41). When the liver suffers from an extended injury, homeostasis between the formation and removal of gut-derived LPS is broken, which may be due to the following reasons. First, sensitivity to gut-derived LPS is increased after the initial liver damage. Second, RES injury leads to hampered detoxification and clearance of gut-derived LPS. Third, gut barrier dysfunction allows more translocation of LPS. Last, bacteria overgrowth and delay of gastrointestinal motility increase production and spillover of gut-derived LPS.

### Gut-Derived LPS Aggravate the Liver Injury

Gut-derived LPS as a cofactor plays a universal role in acute liver injury, which has been demonstrated in acute liver injury models induced by different hepatotoxic agents, including CCl_4_, acetaminophen, alcohol, and D-Gal (29). Under primary liver damage, gut-derived LPS can activate Kupffer cells to release pro-inflammatory mediators, such as TNF-α, interleukins (IL-1 and IL-10), lysosomal enzymes (protease and phosphatase), and superoxide, which aggravate inflammatory responses and necrosis (29). Induction of LPS tolerance protects rats from CCl_4_-induced liver necrosis, and LPS-binding protein also has a protective effect on acute liver injury (29).

In addition to acute liver injury, gut-derived LPS plays a critical role in chronic liver injury. Elevated plasma endotoxin is observed in patients with alcoholic liver disease (ALD) and experimental models of alcoholic liver injury, which can be attenuated by oral antibiotics (42, 43). However, LPS alone fails to mimic ethanol-induced steatosis, but together with ethanol, which is metabolized to acetaldehyde by gut bacteria and intestinal mucosa, leads to hepatocyte steatosis (42). Besides, fibrogenesis usually occurs in the advanced ALD and other chronic liver diseases, such as nonalcoholic fatty liver disease (NAFLD) and chronic hepatitis B (CHB), which can develop into cirrhosis (44). Chronic liver diseases are accompanied by dysbiosis of gut microbiota, which contributes to intestinal dysmotility, inflammation, and mucosal leakage, leading to continuous and excessive liver exposure to gut-derived LPS (45). Toll-like receptor-4 (TLR4) mutation and gut sterilization prevent hepatic fibrosis in mice, revealing that gut-derived LPS contribute to hepatic fibrosis (46). On one hand, when the liver is exposed to increased gut-derived LPS, TLR4 activation of hepatic stellate cells upregulates the production of chemokine (CCL2) and induces chemotaxis of Kupffer cells (47). On the other hand, LPS binding to TLR4 on hepatic stellate cells downregulates transforming growth factor β (TGF-β) pseudoreceptor BAMBI through Myd88- NF-κB-dependent signals, which sensitizes hepatic stellate cells to TGF-β released by Kupffer cells, promoting transdifferentiation of quiescent hepatic stellate cells to activated scar-forming myofibroblasts (46), and myofibroblasts generate extracellular matrix (ECM) materials, including collagen, laminin, and fibronectin. Excessive deposition of ECM will cause aberrant scar formation and fibrosis, which diminishes liver regeneration (48).

In addition, chronic liver diseases are driven by vicious cycles of liver injury, inflammation, repair, and regeneration, which make an opportunity for hepatocellular carcinoma (HCC) development (49). Previous TLR4-deficient, gut-sterilized, germ-free, and LPS-treated animal experiments have evidenced that gut-derived LPS contribute to hepatocarcinogenesis (50, 51). Mechanistically, the LPS-TLR4 pathway contributes to liver tumor promotion by increasing proliferation and preventing apoptosis of non-bone marrow-derived resident liver cells, and in the early phases of HCC, TLR4-dependent secretion of hepatomitogen epiregulin by hepatic stellate cells mediates HCC promotion (51).

The broad roles of gut-derived LPS in liver injury are well established and accepted, but applicating this knowledge to develop an effective treatment remains challenging, which needs further study.

### Gut-Derived LPS Promote Liver Regeneration

Gut-derived LPS also plays an important role in liver regeneration. Restriction of gut-derived LPS by gut bacterial depletion, endotoxin neutralization, and induction of endotoxin tolerance significantly impairs liver regeneration in rats, which is reversed by exogenous LPS supplements (7). In addition, impaired liver regeneration is also observed in germ-free mice receiving partial hepatectomy and in rats simultaneously receiving partial hepatectomy and colon bowel resection (8, 28).

When the liver is subjected to an experimental physical or a chemical injury, gut-derived LPS will pass through the compromised liver and spill into the general circulation, leading to low-grade systemic endotoxemia, which elicits hepatotrophic factors production, such as insulin, glucagon, epidermal growth factor (EGF), vasopressin and triiodothyronine (T3) (6, 52). In addition, gut-derived LPS activates Kupffer cells by binding to TLR-4 for activation of NF-κB and subsequently stimulates the production of TNF-α, which in return activates Kupffer cells to secrete interleukin-6 (IL-6) (53). IL-6 trans-signaling through the soluble IL-6/IL-6R complex induces hepatic stellate cells to produce hepatocyte growth factor (HGF) (54). HGF cooperates with other extrahepatic factors, such as T3, insulin, and EGF, allowing the remnant hepatocytes to overcome cell-cycle checkpoint control to proliferate, which is essential for the priming phase of liver regeneration after partial hepatectomy (53, 54). Finally, when the liver regenerates sufficiently and the phagocytosis function of the Kupffer cell is restored, gut-derived LPS in the portal blood can be detoxified again (6).

**Figure 3:**
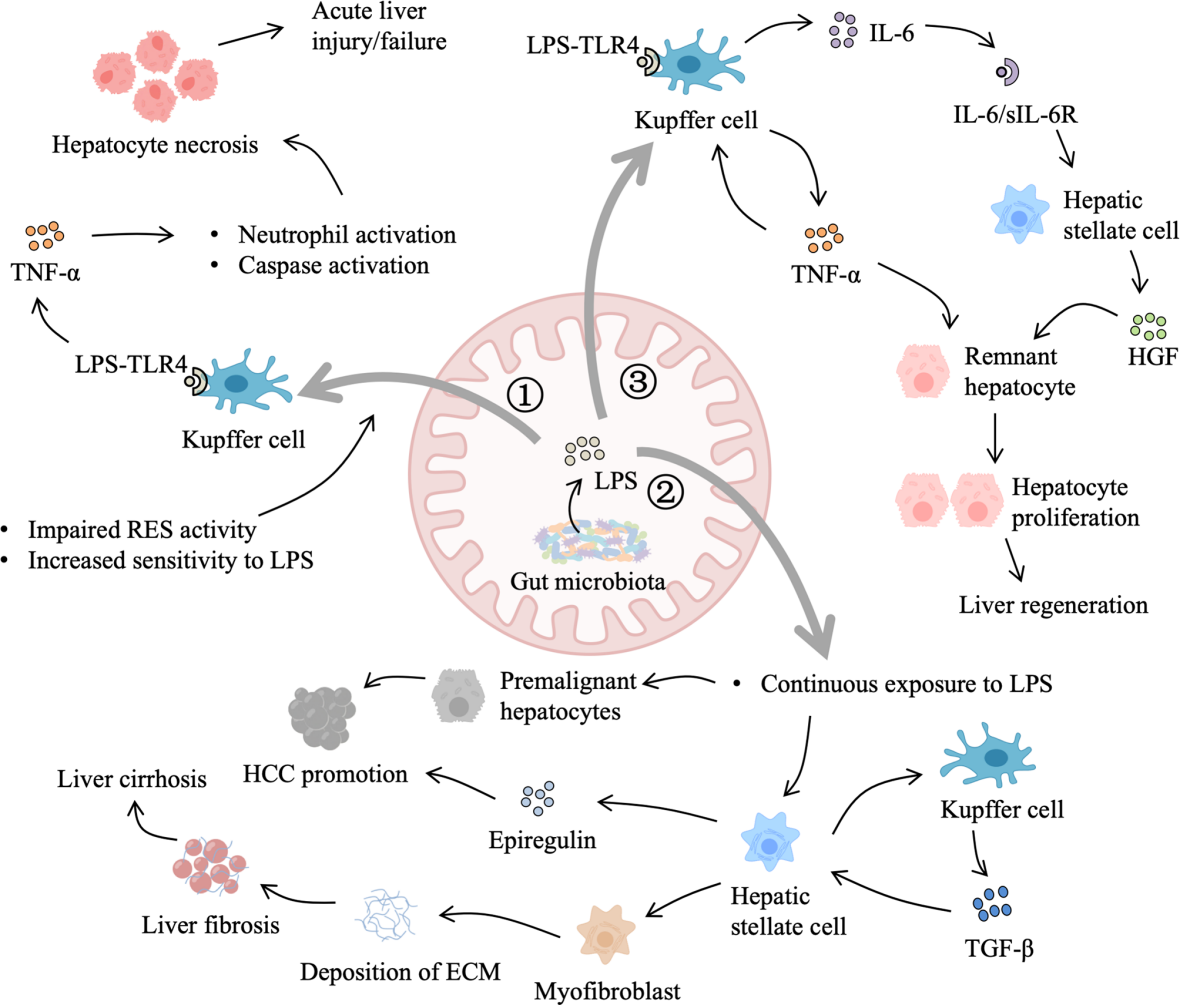
Role of gut-derived LPS in liver injury and regeneration. 1) When the liver suffers from an acute injury, primary damage reduces the activity of the reticuloendothelial system (RES) and increases liver sensitivity to LPS. LPS binding to Toll-like receptor 4 (TLR4) on Kupffer cells triggers the production of tumor necrosis factor-α (TNF-α), which leads to hepatocyte necrosis and aggravates the primary damage. 2) In addition, gut-derived LPS also contributes to chronic liver injury. Dysbiosis of gut microbiota leads to elevated LPS and impaired gut barrier function. Continuous LPS exposure sensitizes hepatic stellate cells to transforming growth factor-β (TGF-β) and promotes the transdifferentiation of hepatic stellate cells into myofibroblasts, resulting in the generation of extracellular matrix (ECM) materials. Excessive deposition of ECM interferes with normal regeneration, leading to liver cirrhosis and hepatocellular carcinoma (HCC) promotion. Besides, LPS accelerates the development of premalignant hepatocytes and stimulates hepatic stellate cells to secret epiregulin, which facilitates HCC promotion. 3) Moreover, after partial hepatectomy, gut-derived LPS activate Kupffer cells to secrete TNF-α, which in return stimulates Kupffer cells to produce interleukin-6 (IL-6). IL-6/IL-6 receptor complex induces the production of hepatocyte growth factor (HGF) by hepatic stellate cells. In addition, gut-derived LPS that escape Kupffer cells spilling into the general circulation elicit systemic hepatotrophic factors production. HGF, TNF-α, and other hepatotrophic factors allow remnant hepatocytes to overcome cell-cycle checkpoint and support liver regeneration.

Therefore, gut-derived LPS are important for liver regeneration as well as liver injury, liver fibrosis, and liver tumors, which may depend on the degree and duration of exposure (**Figure 3**). However, it is difficult to determine the beneficial level of gut-derived LPS for the liver in different scenarios to avoid the deleterious effects of excessive TLR-4 activation. One promising strategy is modulating gut microbiota by probiotics, prebiotics, and perhaps appropriate antibiotics, such as rifaximin, to control gut-derived LPS.

## Gut Microbiota-Associated Bile Acid Metabolism Is Involved in Liver Injury and Regeneration

Bile acids (BAs), produced from cholesterol, are assembled as primary conjugated BAs in the liver and actively transported into the biliary system. A small fraction of BAs circulates from cholangiocytes to the liver through the cholangio-hepatic shunt, while most of them are stored in the gallbladder and released into the duodenum after food intake. Approximately 95% of BAs are reabsorbed *via* the apical sodium-dependent bile acid transporter (ASBT) in the terminal small intestine and return to the liver through the portal vein. Conjugated primary BAs can also be deconjugated by the gut microbiota and escape reabsorption, further dehydroxylated by microbial bioconversion to secondary BAs. A part of secondary BAs is passively absorbed by colonic cells. Spillover of BAs into systemic circulation can be cleared *via* urinary excretion. Bile acids that are lost in urinary and fecal excretion are replenished by hepatic synthesis (55, 56).

**Figure 4:**
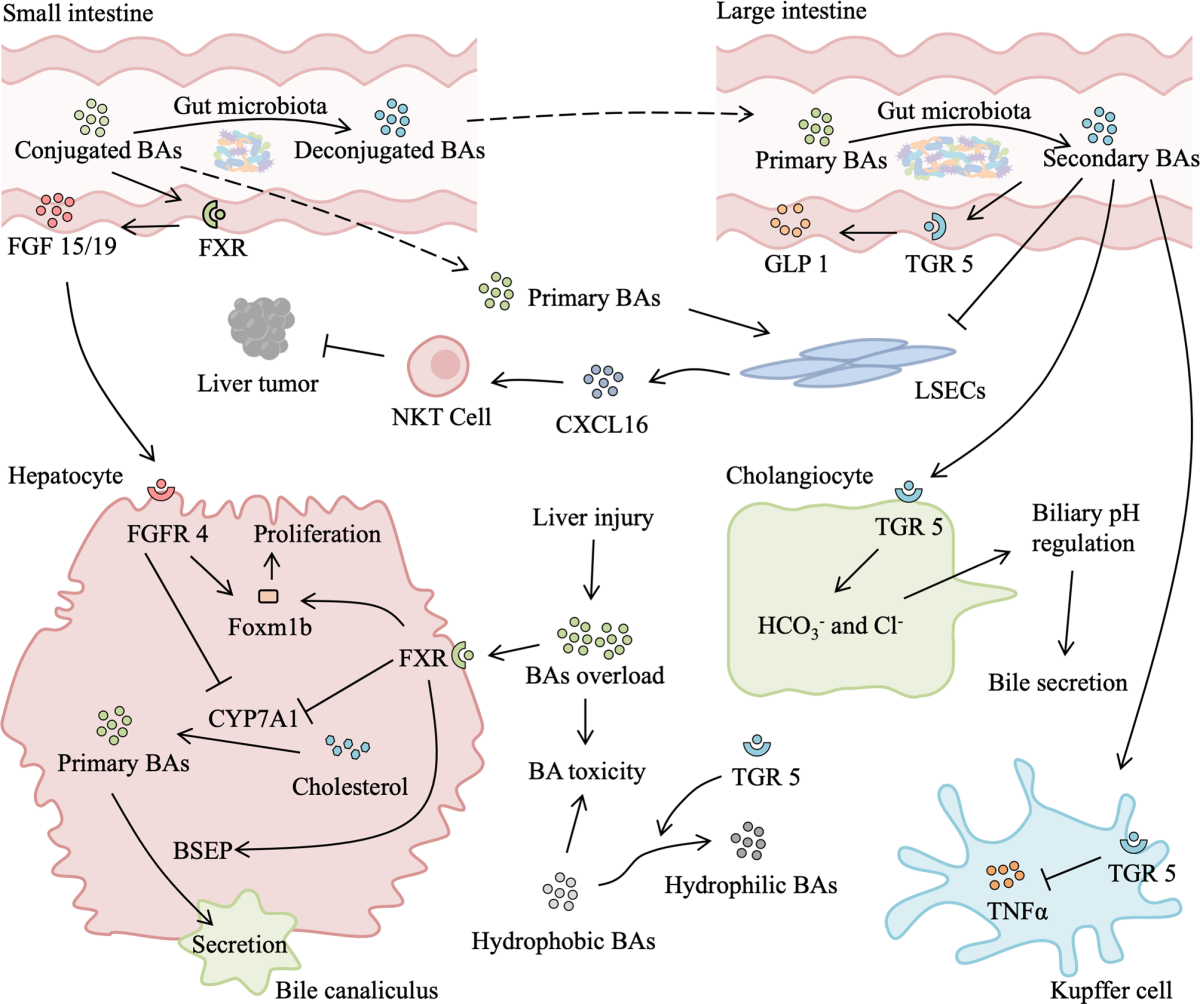
Role of gut microbiota-associated bile acids metabolism in liver injury and regeneration. After a liver injury or partial hepatectomy, bile acids (BAs) absorbed from the intestine suddenly become too high for the remnant liver, leading to secondary liver injury. Excessive BAs binding to hepatic farnesoid X receptor (FXR) inhibits transcription of cytochrome P450 family 7 subfamily A member 1 (CYP7A1), which reduces the production of primary BAs. In addition, activated FXR can promote the secretion of primary BAs into bile canaliculus and upregulate expression of Foxm1b, thus relieving BAs overload and facilitating liver regeneration. Meanwhile, in the small intestine, BAs activate intestinal FXR to secret fibroblast growth factor (FGF) 15/19, which binds to fibroblast growth factor receptor 4 (FGFR4). FGFR4/agonist also induces expression of Foxm1b and improves proliferation. Gut microbiota is responsible for secondary BAs production in the large intestine. Secondary BAs binding to intestinal transforming growth factor 5 (TGR5) elicits secretion of glucagon-like peptide 1 (GLP1), which activates the insulin signaling pathway. Absorbed secondary BAs binding to hepatic TGR5 has an anti-inflammatory effect by suppressing the release of TNF-α from the Kupffer cell. TGR5 activation also promotes BAs secretion by secreting ${\text{HCO}}_{3}^{-}$ and Cl^-^ from cholangiocyte and increasing transformation of hydrophilic BAs from hydrophobic BAs, which reduce BAs load and BAs toxicity-induced liver injury, promoting liver regeneration. Moreover, primary BAs stimulate and secondary BAs suppress the expression of chemokine (C-X-C motif) ligand 16 (CXCL16) by liver sinusoidal endothelial cells (LSECs). CXCL16 is a chemokine that recruits natural killer T (NKT) cells, which suppress liver tumors.

In normal conditions, BAs almost recycle within the enterohepatic circulation, which has important physiological roles in nutrient absorption and biliary secretion of lipids and toxic metabolites, and only traces of BAs escape to the systemic circulation. After partial hepatectomy, BAs that are reabsorbed from the intestine suddenly become too high for the remnant liver, which leads to an abrupt and massive spillover of BAs to the systemic circulation (57, 58). Bile acids overload beyond a certain threshold is deleterious (59). However, BAs are also necessary for normal liver regeneration. Depletion of BAs by cholestyramine (a BA-sequestering resin) leads to suppression of liver regeneration (60). In rats after partial hepatectomy and mice with CCl_4_-induced injury, elevated BAs accelerate liver regeneration, while low levels of BAs impair liver regeneration (60, 61). The similar phenomenon is observed in clinical scenarios, following a major hepatectomy, patients without external biliary drainage have better liver regeneration than those with external biliary drainage (62). Gut microbiota-dependent BA metabolism is likely to participate in liver injury and regeneration by modifying the quality (conjugated vs deconjugated and primary vs secondary BAs) and quantity of the BA pool (**Figure 4**).

### Gut Microbiota-Mediated Deconjugation of BAs Participates in Liver Injury and Regeneration

After partial hepatectomy, to protect the remnant liver and biliary tree from excess BAs, the basolateral uptake and BAs production are decreased and the basolateral efflux and biliary excretion are increased. Deconjugation (removal of the glycine or taurine) by gut microbiota with bile salt hydrolase (BSH) prevents reabsorption of BAs in the small intestine (63). BSH is enriched in the human gut microbiota and mediates bile tolerance (64). Conjugated BAs are transported by sodium taurocholate cotransporting polypeptide (NTCP) and organic anion-transporting polypeptide (OATP) isoform, which are the major uptake transports of BAs in the liver. The mRNA levels of *NTCP*, *OATP1*, and *OATP2* are decreased with the most prominent decrease of *NTCP*, while the protein level of NTCP is markedly decreased during the initial phase of liver regeneration (65). Downregulated NTCP relieves basolateral over uptake of BAs in the liver, but OATP expressing hepatocytes could ensure ongoing basolateral uptake of BAs after partial hepatectomy. However, one study demonstrates that the mRNA and protein levels of NTCP are unchanged after partial hepatectomy (66).

Despite the conflicting effects of partial hepatectomy on NTCP expression, high levels of BAs in the remnant liver are generally accepted. BAs overload inhibits the synthesis of BAs by negative feedback regulation through the nuclear receptor farnesoid X receptor (FXR), which is highly expressed in the liver and ileum (63). Chenodeoxycholic acid (CDCA) is the most potent efficacious ligand of FXR, followed by lithocholic acid (LCA), deoxycholic acid (DCA), and cholic acid (CA) (67). LCA and DCA are secondary BAs transformed from primary BAs by microbial 7α-dehydroxylation, which is a characteristic of *Clostridium* and *Eubacaterium* (68, 69). In the liver, FXR activation induces expression of small heterodimer partner 1 (SHP-1), which can inhibit expression of CYP7A1, the rate-limiting enzyme of BAs synthesis, by reducing the activity of liver receptor homolog 1 (LRH-1) (70). Therefore, BAs overload leads to decreased BAs production by repressing the transcription of the rate-limiting enzyme in BAs synthesis. In addition, increased basolateral efflux and biliary excretion of BAs reduce BAs concentrations in hepatocytes, which protects the liver from BA-induced liver injury. Conjugated BAs are primarily secreted into bile *via* the canalicular bile salt export pump (BSEP) (55). The expression of BSEP is increased from days 1 to 3 after partial hepatectomy, which depends on the activation of FXR (60). Thus, the remnant liver copes with the BAs overload *via* FXR to maintain normal BAs levels, including both repressions of synthesis and induction of export.

Besides, hepatic FXR plays a critical role in the expression of Foxm1b, a key regulator of the hepatic cell cycle, promoting liver regeneration after either partial hepatectomy or CCl_4_-induced liver injury (71). Moreover, compared with liver-specific FXR knock-out (KO) mice, conventional FXR KO mice show significantly decreased liver regeneration response at 36 h and 72 h after partial hepatectomy, suggesting that FXR activation in other tissues also contribute to liver regeneration (71).

Intestinal FXR may participate in the promotion of liver regeneration through secreting fibroblast growth factor (FGF) 15 in mice or FGF19 in humans. Trans-enterocytic BAs flux from the intestinal lumen to the basolateral side drives FXR-dependent FGF15 synthesis in the ileum (72). FGF15 reaches the liver and regulates BA homeostasis by an FGFR4-dependent activation of c-Jun N-terminal kinase (JNK) and extracellular signal-regulated kinase (ERK) pathways, leading to transcriptional inhibition of CYP7A1 in hepatocytes (72). Therefore, BAs-mediated intestinal FXR-dependent FGF15 production appears as a necessary gut-derived signal for liver protection after partial hepatectomy by maintaining BAs homeostasis. Besides, FGF15 may directly contribute to liver regeneration by stimulating the proliferation of hepatocytes and cholangiocytes (73). Mechanistically, the FGF15-FGFR4-STAT3 signaling pathway, which is required for Foxm1 transcription and cell cycle progression, controls hepatocyte proliferation in the regenerating liver (74). Furthermore, it should be noted that FXR is expressed in the kidney at high levels and in the thymus, spleen, ovary, testes, heart, and eyes at lower levels (63). Increased systemic BAs after partial hepatectomy may also act on these tissues and organs in an FXR-dependent manner and indirectly affect liver regeneration, which needs further exploration.

Taken together, gut microbiota-mediated deconjugation improves the reabsorption of BAs and the abundance of colonic primary conjugated BAs, which may induce protective as well as proliferative cascades in the damaged liver by initiating FXR-dependent responses.

### Gut Microbiota-Dependent Production of Secondary BAs Facilitates Liver Regeneration

Gut microbiota dehydrogenates primary BAs to secondary BAs mainly in the colon. Secondary BAs are the most efficient agonists of Taketa G-protein-coupled receptor 5 (TGR5) (75). TGR5 is ubiquitously expressed in many tissues, including the liver, gallbladder, intestine, brown and white adipose tissue, skeletal muscle, and so on (63). In the liver, TGR5 is highly expressed in cholangiocytes, Kupffer cells, and endothelial cells but weakly or not expressed in hepatocytes (76). Therefore, TGR5-dependent protective effects against BAs overload are likely due to other mechanisms rather than regulation of BAs synthesis. TGR5 regulates BA size and composition by reducing hydrophobicity and increasing secretion. A shift towards a more hydrophobic BAs pool is associated with inhibition of liver regeneration (77). TGR5-KO mice have a more hydrophobic BAs composition and hydrophobic BAs accumulation in the liver leads to toxic injury, which is alleviated by a BA resin enriched diet (58). In addition, TGR5 promotes cystic fibrosis transmembrane conductance regulator (CFTR)-dependent Cl^-^ secretion and BAs uptake into biliary epithelia and reduces biliary bile acid concentrations (78). TGR5-dependent increased output of biliary $HCO_{3}^{-}$ and Cl^-^ after partial hepatectomy also enhances bile secretion, which prevents the remnant liver from BAs-induced toxicity (58). Furthermore, the production and release of the cytokine after partial hepatectomy are crucial for normal liver regeneration. It has been demonstrated that the immunosuppressive effect of secondary BAs on macrophage is mediated by TGR5 (79), which inhibits LPS-induced expression of cytokines and reduces liver injury (80), by suppressing NF-κB transcription activity and its target gene expression (81). Therefore, gut microbiota-controlled activation of TGR5 may contribute to liver regeneration by regulating hepatic inflammatory response and limiting hepatocyte necrosis after partial hepatectomy. Moreover, TGR5 in skeletal muscle and brown adipose tissue promotes energy expenditure through iodothyronine deiodinase 2 enzyme (DIO2), which converts inactive thyroxine into active thyroid hormone (63). TGR5 in colonic L cells mediates synthesis and secretion of intestinal glucagon-like peptide-1 (GLP-1), which could stimulate insulin secretion (82). Thyroid hormone and insulin cooperated with other growth factors allow the hepatocyte to overcome cell-cycle checkpoint control, initiating and regulating liver regeneration (53).

Gut microbiota-dependent conversion of primary BAs to secondary BAs is also involved in the development of liver cancer (83). Primary BAs stimulate, whereas secondary BAs suppress, the expression of chemokine (C-X-C motif) ligand 16 (CXCL16) by liver sinusoidal endothelial cells, which induces accumulation of hepatic CXCR6+ natural killer T (NKT) cells and production of interferon-γ, inhibiting both primary and metastatic liver tumors.

In general, gut microbiota-dependent production of secondary BAs may reduce the inflammatory response and promote liver regeneration by TGR5-dependent regulation of the BA pool and production of T3 and GLP-1. Elucidating ways to fine-tune gut microbiota-BAs-host interaction is a promising strategy to assist normal liver regeneration.

## Gut Microbial Metabolites Short-Chain Fatty Acids and Indoles May Assist Liver Regeneration in a Similar Way

### Short Chain Fatty Acids May Assist Liver Regeneration

Short-chain fatty acids (SCFAs) produced by gut microbial fermentation have multiple physiological functions (84). The most abundant SCFAs in the gut are acetate, propionate, and butyrate (85, 86). Bacteroidetes produce acetate and propionate, and Firmicutes are the primary butyrate producers (87–90).

The intestinal tract is the major site of SCFAs production and the biological concentration gradient falls from the gut to the peripheral tissues. Butyrate is largely metabolized in the intestinal epithelium, and the rest is degraded in the liver (91, 92). Most propionate is degraded in the liver, and a substantial portion of acetate passes into the systemic circulation (92, 93). SCFAs function as extracellular agonists for G-protein-coupled receptor (GPR) 41 and GPR43 (94). Butyrate, but not acetate or propionate, also activates the GPR109A (95). GPR41, GPR43, and GPR109A are expressed by intestinal epithelial cells and immune cells (84). Stimulation of GPRs by SCFAs activates ERK1/2, c-JNK, and p38/mitogen-activated protein kinases (MAPKs) (96). In addition, butyrate broadly affects transcription by activating histone acetyltransferases (HATs) and suppressing nuclear class I histone (HDACs) (84). Butyrate can also act as an extracellular agonist activating peroxisome proliferator-activated receptor γ (PPAR-γ), which plays anti-inflammatory effects (97, 98).

SCFAs are important substrates for the integrity of the epithelial barrier, which limits pro-inflammatory load to the liver. Butyrate enhances gut barrier function by increasing the expression of claudin-1 and zonula occludens-1 (ZO-1), decreasing LPS translocation and inhibiting downstream inflammatory responses (99, 100). Therefore, SCFAs may indirectly affect liver injury and regeneration through maintaining gut barrier function.

In addition, SCFAs play potential roles in metabolism homeostasis during liver regeneration. Acetate, propionate, and butyrate regulate hepatic glucose and lipid homeostasis in a PPAR-γ dependent manner (101). Acetate and propionate inhibit adipocyte lipolysis *via* GPR43, which reduces free fatty acid (FFA) flux to the liver and ameliorates the deterioration of glucose homeostasis caused by fatty liver (102, 103). Propionate and butyrate have metabolic benefits through activating gluconeogenesis gene expression (104). Moreover, SCFAs stimulate gut hormone production of GLP-1 and peptide YY (PYY), improving the metabolic phenotype (105). Butyrate inhibits NF-κB activation in lamina propria macrophages and reduces the responsiveness of lamina propria macrophages to commensal bacteria (106, 107). Butyrate alleviates ischemia-reperfusion liver injury by preventing NF-κB activation and reducing inflammatory factors production (108). Propionate and butyrate have potential roles in the production and function of regulatory T cells *via* inhibiting histone deacetylase (HDAc) (109, 110).

**Figure 5:**
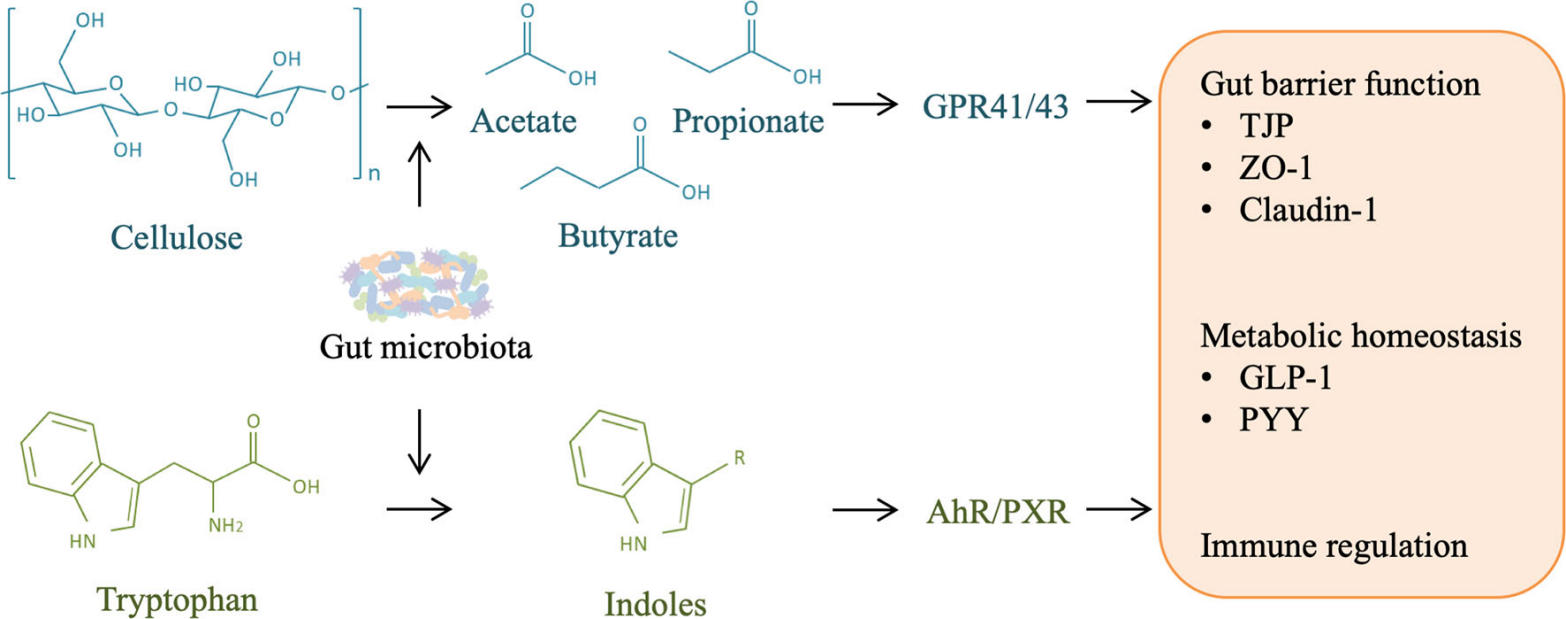
Role of other microbial metabolites in liver injury and regeneration. Gut microbiota is responsible for the production of short-chain fatty acids (SCFAs) and indoles from cellulose and tryptophan in food-intake, respectively. SCFAs can activate host G-protein-coupled receptor (GPR) 41/43, and indoles can activate aryl hydrocarbon receptor (AHR)/pregnane X receptor (PXR), contributing to gut barrier protection, metabolism homeostasis, and immune regulation, which may facilitate liver regeneration by alleviating liver injury.

These studies highlight the interaction between gut microbiota-derived SCFAs and the immune and metabolic homeostasis of the host, which may play an important role in liver injury and regeneration (**Figure 5**).

### Indoles May Assist Liver Regeneration

Tryptophan is one of the essential amino acids and must be supplied by dietary uptake. The majority of tryptophan is absorbed in the small intestine, and a significant fraction may also reach the colon. Intestinal tryptophan metabolism follows three major pathways: (1) the kynurenine pathway in epithelial and immune cells through indoleamine 2,3-dioxygenase 1 (IDO1); (2) the serotonin pathway in enterochromaffin cells through tryptophan hydroxylase 1 (TpH1); and (3) direct transformation by commensal bacteria into indole and indole derivatives (111). Numerous bacterial species can metabolize tryptophan, which has been described in a previous review (112). Indole is the most abundant metabolite of gut microbial tryptophan, which is followed by indol-3-acetic acid (IAA) and indole-3-propionic acid (IPA), in adults (112). Indole and many indole derivatives, such as IAA, IPA, indole-3-aldehyde (IAld), tryptamine (TA), 3-methylindole (skatole), and indoxyl-3-sulfate (I3S), are endogenous ligands of aryl hydrocarbon receptor (AhR) (113). AhR is initially well-known for its major role in the metabolism and elimination of environmental toxicants, but it also serves as a key transcription factor controlling many critical cellular functions and organ homeostasis (114).

Given the production site, the role of gut microbial tryptophan metabolism is preponderant in intestinal AhR activity. Activation of AhR in the gut improves intestinal barrier function by decreasing gut permeability and mucosal inflammation (83). Indole upregulates the expression of genes involved in the maintenance of epithelial-cell tight-junction resistance (115, 116). Indoleacrylic acid (IA) improves intestinal epithelial barrier function by promoting goblet cell differentiation and mucus production, which is possibly mediated by AhR activation (117). Moreover, in the context of indole, IPA also regulates intestinal barrier function by acting as a ligand for the pregnane X receptor (PXR) (118). These studies suggest that tryptophan metabolites enhance the intestinal epithelial barrier function by AhR and PXR signaling pathways, which decreases translocation of gut-derived LPS and then could participate in liver injury and regeneration. Similar to SCFAs, indole is also able to modulate the secretion of GLP-1 from colonic L cells and influence host metabolism in an AhR-dependent manner (119, 120). GLP-1 has an important role in glucose homeostasis and liver function, which is probably involved in liver regeneration.

In addition, AhR is expressed by many immune cells and plays an important role in the regulation of immune response in health and disease (113). Gut microbiota-derived IAA and TA reduce inflammatory responses in macrophages and hepatocytes (121), suggesting that gut microbial tryptophan metabolites could regulate immune responses in the liver as well, which indicates their potential roles in liver injury and regeneration. However, the exact effects of gut microbial tryptophan metabolites on liver regeneration still need further research (**Figure 5**).

## Conclusion

Mechanisms of liver regeneration are complex. The crosstalk between gut microbiota and liver allows that gut-derived signals are orchestrated in liver injury and regeneration. Gut-derived LPS and gut microbiota-associated bile acid metabolism appear to have multiple effects on liver injury and regeneration, while SCFAs and tryptophan metabolites produced by gut microbiota have potential benefits in liver regeneration. A comprehensive understanding of these roles of gut-derived signals in liver injury and regeneration will enable the further development of rational specific therapies to either directly improve liver regeneration or prevent complications that appear in the process of liver regeneration. The role of gut-derived signals in animal models of liver regeneration has been briefly described in this review, but lessons learned from animal models still need to be confirmed in more clinical settings, such as chronic liver injury with abnormal liver architecture and advanced liver fibrosis, in which normal regeneration of compromised liver is expected. However, gut-derived signals have the promise to motivate translational and interventional studies of liver injury and regeneration.

## Author Contributions

ZZ managed the literature research and wrote the first draft of the manuscript. BW critically reviewed the manuscript and provided valuable discussions and criticisms. All authors contributed to the article and approved the submitted version.

## Funding

This study was supported by National Natural Science Foundation of China [grant numbers 82170668 and 81790633], Sino-German Center for Research Promotion [grant numbers GZ1546], and CAMS Innovation Fund for Medical Sciences [grant numbers 2019-I2M-5-045].

## Conflict of Interest

The authors declare that the research was conducted in the absence of any commercial or financial relationships that could be construed as a potential conflict of interest.

## Publisher’s Note

All claims expressed in this article are solely those of the authors and do not necessarily represent those of their affiliated organizations, or those of the publisher, the editors and the reviewers. Any product that may be evaluated in this article, or claim that may be made by its manufacturer, is not guaranteed or endorsed by the publisher.
